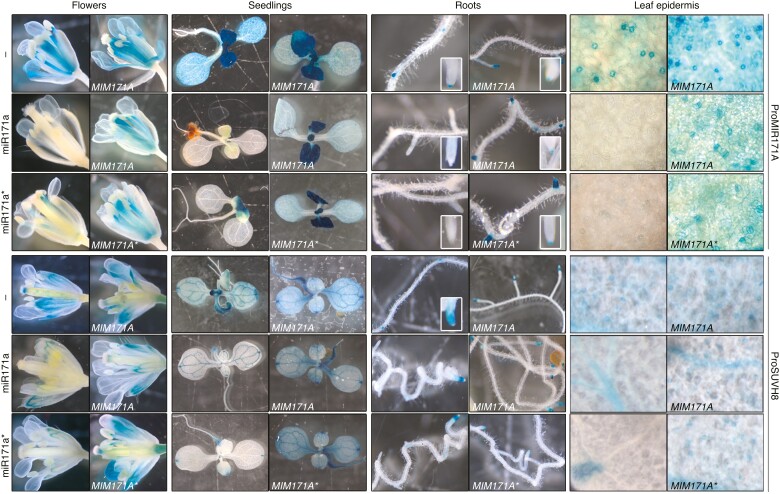# Correction to: Tissue-Specific Silencing of Arabidopsis *SU*(*VAR*)*3-9 HOMOLOG8* by miR171a

**DOI:** 10.1093/plphys/kiae410

**Published:** 2024-08-16

This is a correction to: Pablo A. Manavella, Daniel Koenig, Ignacio Rubio-Somoza, Hernán A. Burbano, Claude Becker, Detlef Weigel, Tissue-Specific Silencing of Arabidopsis SU(VAR)3-9 HOMOLOG8 by miR171a, Plant Physiology, Volume 161, Issue 2, February 2013, Pages 805–812, https://doi.org/10.1104/pp.112.207068

A duplication occurred during preparation of Figure 6, with an image of the ProMIR171A::GUS x MIMI171A (Row 1, Column 6) control cross duplicated in the panel that was supposed to show another control cross, ProSUVH8::GUSmiR171a* x MIMI171A* (Row 6, Column 6). The erroneously duplicated image meant to show, as a control, the reversion of the silencing phenotype in nascent secondary root tips by a mimicry construct designed to titrate the endogenous miR171a*. The same suppression of miR171a*-triggered silencing of ProSUVH8::GUSmiR171a* by MIM171A* was also shown for flowers, seedlings, and epidermis in the same figure (Row 6, Columns 2, 4, and 8). The authors revised all the original photographs and prepared corrected versions with the appropriate image corresponding to the roots of ProSUVH8::GUSmiR171a* x MIMI171A*. The authors apologize for this inadvertent error. This correction does not alter the result, the figure legend nor the main text, and it does not change the conclusions of the paper. All original raw pictures corresponding to this figure have been now uploaded to a public repository (Zenodo doi: 10.5281/zenodo.11635603).

**Figure:**